# Effectiveness of endoscopic vacuum therapy as rescue treatment in refractory leaks after gastro-esophageal surgery

**DOI:** 10.1007/s13304-020-00935-y

**Published:** 2020-11-30

**Authors:** Carlo Alberto De Pasqual, Valentina Mengardo, Francesco Tomba, Alessandro Veltri, Michele Sacco, Simone Giacopuzzi, Jacopo Weindelmayer, Giovanni de Manzoni

**Affiliations:** 1 General and Upper GI Surgery Division, University of Verona, Piazzale A. Stefani 1, 37124 Verona, Italy; 2 Department of Emergency Surgical Endoscopy, Azienda Ospedaliera Universitaria Integrata, Piazzale A. Stefani 1, 37124 Verona, Italy

**Keywords:** E-VAC, Refractory anastomotic leak, Upper-GI surgery

## Abstract

The treatment of leak after esophageal and gastric surgery is a major challenge. Over the last few years, endoscopic vacuum therapy (E-VAC) has gained popularity in the management of this life-threatening complication. We reported our initial experience on E-VAC therapy as rescue treatment in refractory anastomotic leak and perforation after gastro-esophageal surgery. From September 2017 to December 2019, a total of 8 E-VAC therapies were placed as secondary treatment in 7 patients. Six for anastomotic leak (3 cervical, 1 thoracic, 2 abdominal) and 1 for perforation of the gastric conduit. In 6 cases, E-VAC was placed intracavitary; while in the remaining 2, the sponge was positioned intraluminal (one patient was treated with both approaches). A total of 60 sponges were used in the whole cohort. The median number of sponge insertions was 10 (range: 5–14) with a median treatment duration of 41 days (range: 19–49). A complete healing was achieved in 4 intracavitary (67%) and in 1 intraluminal (50%) E-VAC. We observed only one E-VAC-related complication: a bleeding successfully managed endoscopically. E-VAC therapy seems to be a safe and effective tool in the management of leaks and perforations after upper GI surgery, although with longer healing time when it is used as secondary treatment.

## Introduction

Anastomotic leak is one of the most feared complication after upper gastro-intestinal (UGI) surgery, with an incidence ranging between 5 and 30% and an associated mortality of 20–50% [1, 2].

Optimal treatment of the leak requires cleaning of the abscess and avoidance of further contamination through the digestive tract. Therapeutic strategies range from reoperation to percutaneous drain placement and endoscopic stent placement [3]. Endoscopic stent placement is, nowadays, the preferred procedure in stable patients, but it is burdened by a significant risk of complications (perforation, bleeding and stent migration) [3, 4]. Moreover, after the stent is placed, an additional percutaneous drain is often necessary to drain the excluded abscess [5]. Endoscopic vacuum therapy (E-VAC) has emerged over the last few years as an alternative to treat leak and perforations. First introduced in 2003 for the management of anastomotic leak after rectal surgery [6], its use has been successfully extended to the treatment of several different types of gastrointestinal perforations [7, 8]. The negative pressure applied through the sponge allows an efficient drainage of the collections, thus providing an optimal control of the septic source. Moreover, it accelerates the healing process of the wound by improving tissue granulation and vascularization [9].

At present, literature on E-VAC after UGI surgery is still limited to small series and case reports [10] and therefore, several technical factors, such as the optimal sponge placement (intracavitary versus intraluminal), the intensity of negative pressure and the interval between sponge changes, are not standardized. Moreover, these articles focused on E-VAC as primary treatment, while no data have been published on long-lasting defects after the failure of a primary treatment.

In this study, we described our initial experience of E-VAC therapy as rescue treatment in the management of UGI leaks resistant to another treatment.

## Methods

### Patients cohort

We considered all the patients who underwent surgery for both malignant and benign disease at our Institution and patients referred to our Department for the treatment of post-operative leaks or perforations from September 2017 to December 2019. Among these, patients who were treated with E-VAC therapy for esophageal or gastric anastomotic leak and gastric conduit perforation were included in the study.

### Data collection and definitions

Demographic and clinical data were collected from our prospectively maintained Institutional database. Additional data on E-VAC treatment (number of insertions, duration of the treatment and procedure-related complications) were retrospectively collected from the patients’ medical record. Number of procedures was defined as the total number of endoscopies including sponge insertion and changes. We defined procedure-related complication as any adverse event related with sponge placement or removal.

Anastomotic leak was defined according to the Esophagectomy Complications Consensus Group (ECCG) and Gastrectomy Complications Consensus Group (GCCG) classification as “a full thickness gastrointestinal defect involving esophagus, anastomosis, or staple line irrespective of timing and clinical presentation” [1, 11]. Gastric conduit perforation was defined as full thickness gastric tube wall defect irrespective of timing and clinical presentation.

### Leak detection and definition of therapeutic strategy

In the study period, no routine radiologic examination was performed to detect silent leak. Anastomotic leak and perforations were clinically suspected when patients presented fever, leukocytosis, CRP elevation and neck erythema in case of cervical anastomosis. The diagnosis was confirmed using CT scan with oral contrast. An emergency endoscopy was then performed to define the site and size of the leak and the abscess. Therapeutic strategy was tailored on patient’s clinical condition and leak features. The decision of beginning E-VAC therapy was discussed between surgeon and endoscopist.

### E-VAC treatment

All the endoscopic procedures were performed, under conscious sedation, by two experienced endoscopists using the Eso-SPONGE^®^ System (B. Braun Melsungen AG, Melsungen, Germany). The endoscopic procedure consisted of 7 steps:

1. Endoscopic assessment of the leak site and of the wound cavity; the polyurethane foam was, therefore, modeled according to the cavity size in case of intracavitary placement;
2. Debridement and irrigation of the cavity;
3. Positioning of the overtube. In case of intracavitary approach, the overtube was placed into the wound cavity; when the intraluminal treatment was chosen, the overtube was placed nearby the wall defect;
4. Sponge placement into the cavity/lumen through the overtube and overtube removal;
5. Trans-nasal channeling of the drain using a naso-gastric tube;
6. Endoscopic evaluation of the sponge correct positioning
7. Connection of the drain tube to Pleur-evac^®^ Chest Drainage System and activation of the vacuum pump (settings: 35 mmHg, continuous suction).

The E-VAC was changed every 3–4 days and the treatment was continued until the leak was closed and healing was confirmed by an oral contrast swallow (success). E-VAC was suspended (failure) in case of patient’s intolerance or no signs of leak improvement or deterioration of the patient’s clinical conditions.

## Results

During the study period, we performed 80 esophagectomies and 221 gastrectomies for cancer and 9 reconstructions after caustic ingestion (8 colonic interpositions and 1 gastroplasty with cervical anastomosis). Anastomotic leak or conduit perforation rate was 11.2% (9/80) after esophagectomy, 2.2% (3/221) after gastrectomy and 55.5% (5/9) after reconstruction for caustic ingestion. Among these 17 patients, 5 underwent E-VAC therapy after failure of another conservative treatment. Moreover, 2 patients with anastomotic leak after total gastrectomy were transferred to our Department and treated with E-VAC therapy.

### Patient characteristics

A total of 6 patients were treated for anastomotic leak: 3 cervical, 1 thoracic and 2 abdominal. One patient was treated for gastric conduit perforation after an Ivor Lewis esophagectomy. Table 1 summarized patients’ features and the details of postoperative leak or perforation. Median leak detection was on post-operative day (POD) 7 (range 4–255). We observed 2 cases of delayed complications. One patient developed a cervical anastomotic leak on POD 255 after reconstruction for caustic ingestion, probably caused by a vascular ischemia. Another patient had a regular postoperative course after Ivor Lewis esophagectomy but was readmitted on POD 38 for a small (3 mm) full thickness perforation of a prepyloric ulcer.

**Table 1:** Patients characteristic and details of postoperative leak or perforation

Characteristics	Patients (tot 7)
Sex, male	6
Age, median (range)	60 (53–72)
ASA	
1–2	4
3–4	3
Type of surgery	
Retrosternal esophago-gastroplasty after caustic ingestion	1
Mc Kewon esophagectomy for cancer	2
Ivor Lewis esophagectomy for cancer	2
Total Gastrectomy for cancer	2
Site of leak	
Cervical esophago-gastric anastomotic leak	3
Thoracic esophago-gastric anastomotic leak	1
Abdominal esophago-jejunal anastomotic leak	2
Intrathoracic gastric tube perforation	1
POD leak detection, median (range)	7 (4–255)
Defect size	
< 1 cm	1
1–2 cm	3
> 2 cm	1
Complete dehiscence	2
Abscess size	
< 5 cm	1
6–10 cm	4
> 10 cm	2

*POD* post-operative day

### Primary treatments

In our series, E-VAC treatment was always considered after the failure of another treatment, as reported in Table 2. The 3 patients with a cervical leak were initially managed with bedside opening of the cervical wound and conservative treatment (nil per os and naso-gastric tube nearby the defect).

**Table 2:** Treatments before starting E-VAC therapy

Patient	Defect site	Primary treatment	Primary treatment outcome	Secondary Treatment	Secondary treatment outcome	POD E-VAC	Time diagnosis-E-VAC (days)
1	Cervical leak	Conservative^a^ + wound opening	No improvement after 60 days			313	58
2	Abdominal leak	Conservative^a^	Clinical worsening after 20 days			39	32
3	Cervical leak	Conservative^a^ + wound opening	Clinical worsening after 30 days; increase of leak size^c^			37	30
4	Abdominal leak	Surgery: primary closure + drain	Recurrence after 10 days^b^	OVESCO^d^	Recurrence after 7 days^b^	27	21
5	Thoracic leak	Conservative^a^ + percutaneous drain	No improvement after 15 days			20	16
6	Gastric conduit perforation	Surgery: primary closure + drain	Recurrence after 8 days^b^	intraluminal E-VAC^e^	No improvement after 13 days	81	43
7	Cervical leak	Conservative^a^ + wound opening	Increase of leak size after 29 days^c^			29	22

^a^Conservative treatment: nil per os, naso-gastric tube placed near the defect, antibiotics

^b^Recurrence was suspected in case of increase of drain output and/or change of drain quality, and confirmed with CT scan with oral contrast and endoscopy

^c^Increase of leak size assessed with endoscopy

^d^OVESCO: Over-the-scope clip

^e^Details on E-VAC therapy are provided in Table 3

The patient with a thoracic leak after Ivor Lewis esophagectomy required a percutaneous drain placement in the abscess, but after 15 days of conservative treatment, no improvement was observed at the follow-up endoscopy.

Among the 2 patients with abdominal leak after total gastrectomy, one, initially treated in another hospital, had a type III leak that required surgery with primary closure of the defect and drain of the abscess. The patient was then transferred to our Department, due to a progressive worsening of the clinical condition, and endoscopy revealed a leak with a large but well drained cavity. The endoscopist tried to seal the defect with an Over-the-Scope clip but after 7 days, it was displaced without improvement of the leak. The latter patient presented a small leak that was initially treated conservatively.

The patient with the gastric conduit perforation presented a thoracic empyema that required an emergency surgery with toilette of the chest cavity and primary closure attempt. On POD 8, a small residual defect was detected with a soluble contrast swallow test and a first attempt of intraluminal E-VAC was made.

### E-VAC treatment

**Fig. 1:**
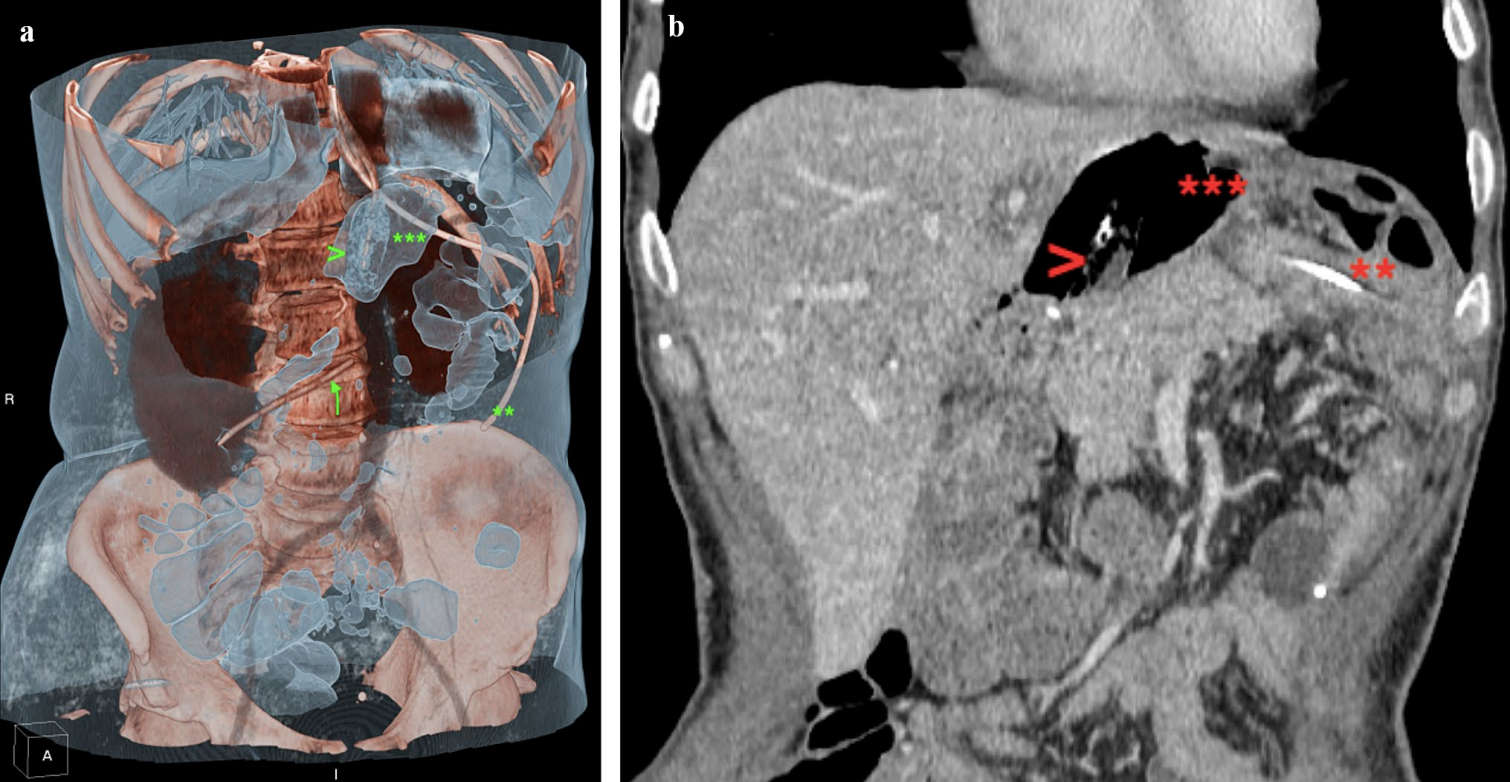
3D CT reconstruction (Fig. 1a) and CT scan (Fig. 1b) of esophagojejunal anastomotic leak (patient *n* 2) with esosponge in place. > Sponge; **nasojejunal feeding tube; ***wound cavity; arrow: abdominal drain

**Fig. 2:**
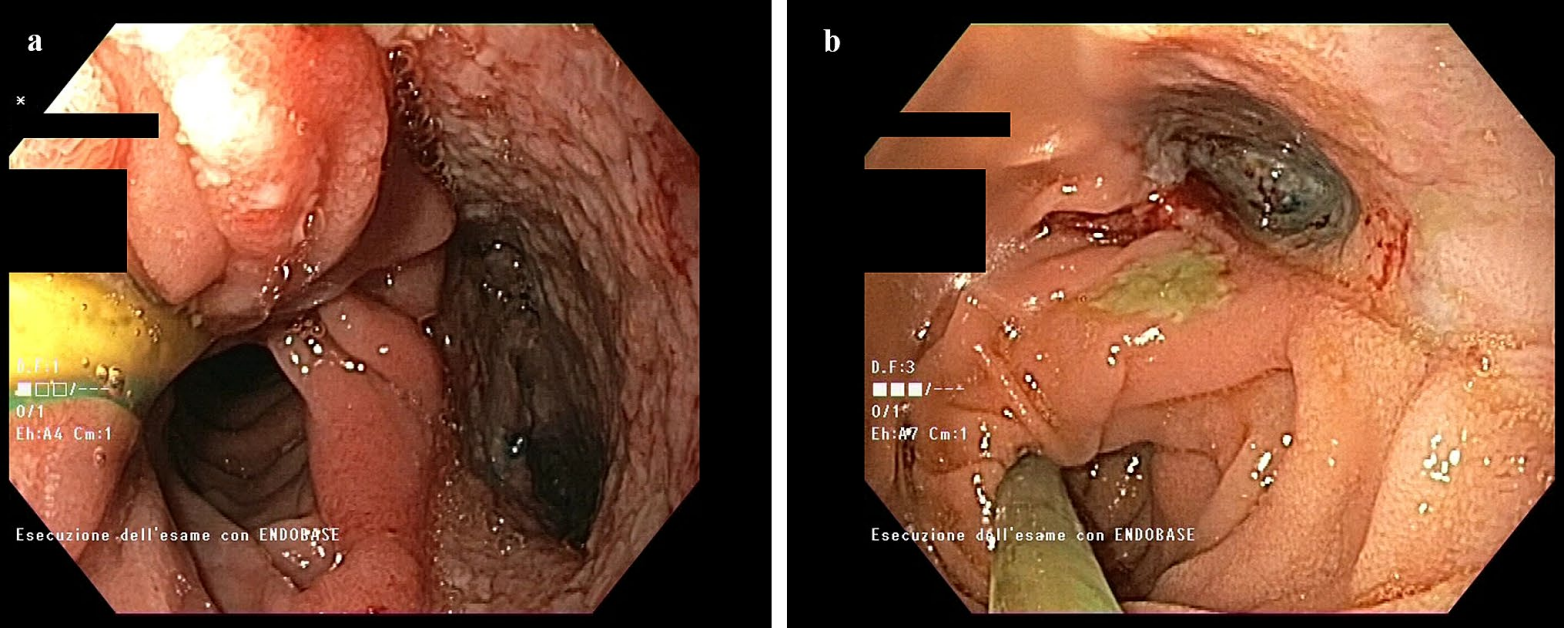
Endoscopic view of esophagojejunal anastomotic leak (patient 4) at the first esosponge replacement (Fig. 2a) and at the end of the treatment (Fig. 2b)

During the study period, we performed 6 intracavitary E-VAC and 2 intraluminal E-VAC treatments in 7 patients, with an overall number of 60 procedures. Details on E-VAC treatment are presented in Table 3 and an example of CT reconstruction of esophagojejunal anastomotic leak with esosponge in place is provide in Fig. 1. Median time between surgery and first E-VAC placement was 37 days (range 20–313), while median time from leak diagnosis and the beginning of E-VAC treatment was 30 days (range 16–58). In the entire cohort, the treatment required a median of 19 days (range 4–49) and 5 procedures (range 1–14) per patient. A complete healing of the leak was achieved in 4 cases of intracavitary (67%) and in 1 case of intraluminal (50%) treatment (Fig. 2). Considering only the patients successfully treated with E-VAC, the median number of procedures was 10 (5–14) with a median treatment duration of 41 days (19–49).

**Table 3:** E-VAC treatment details

Patient	Position	N procedures	Treatment duration (days)	Complications	Success	Cause of failure	Other treatments
1	Intracavitary	5	19	Bleeding	No	Bleeding	Conservative^a^
2	Intracavitary	8	29	No	Yes		
3	Intracavitary	1	4	No	No	Neck pain	Conservative^a^
4	Intracavitary	10	41	No	Yes		
5	Intracavitary	14	49	No	Yes		
6	Intraluminal	3	13	No	No	No local improvement, sepsis	Surgery^b^; intracavitary E-VAC
6	Intracavitary	14	46	No	Yes		
7	Intraluminal	5	19	No	Yes		
Tot		Median 6.5	Median 24	12.5%	62.5%		

^a^Conservative treatment: nil per os, naso-gastric tube placed near the defect, antibiotics

^b^Surgery: thoracoscopy with closure of the defect, pleural toilette and drain placement

**Fig. 3:**
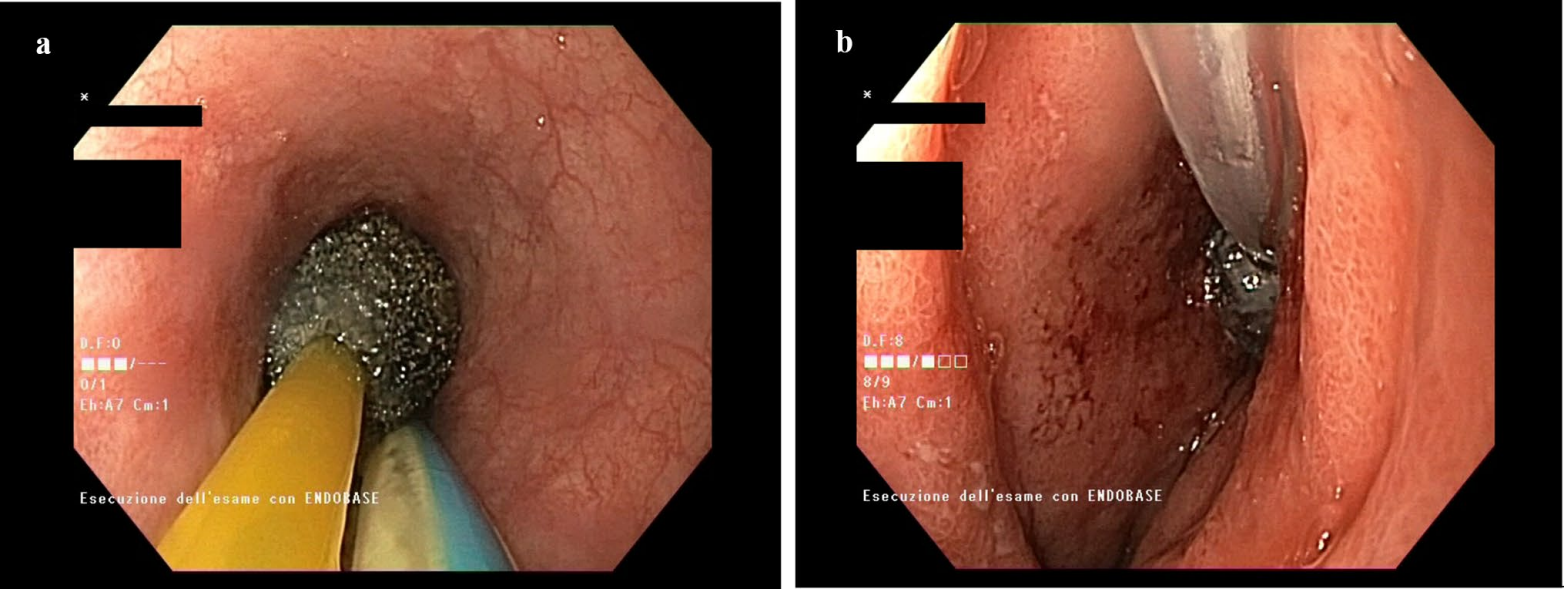
Different sponge positioning. Figure 3a intraluminal sponge placement. Figure 3b intracavitary sponge placement

As above mentioned, the patient with a small residual defect after primary closure of the gastric conduit perforation was initially treated with intraluminal E-VAC therapy. After 13 days and 3 sponge replacements, we did not observe significant improvements and, due to a further deterioration of the patient’s clinical condition, the treatment was stopped. The patient underwent a second surgical operation with thoracic toilette and a new direct closure of the wall defect. Unfortunately, this treatment failed too, and, at the endoscopy, a small (< 1 cm) defect with a large cavity (> 10 cm) was detected. We decided to perform a secondary attempt with an intracavitary E-VAC but, to allow the extraluminal placement of the sponge, a gentle dilation of the leak was done (Fig. 3). After 46 days and 14 procedures, a complete healing of the cavity and a closure of the leak were obtained.

In our experience, we reported only one E-VAC-related complication: one patient with a cervical leak presented an intracavitary bleeding during the sponge replacement that was successfully managed endoscopically. However, E-VAC treatment was interrupted to avoid further complications. E-VAC therapy failed in another patient with cervical leak that was unable to continue the therapy after 4 days for severe neck pain.

Within 90 days after the first E-VAC placement, only 1 patient with a cervical anastomotic leak developed a stenosis, successfully treated with endoscopic dilatations. No 90-day mortality was observed.

## Discussion

Anastomotic leak and perforations are major complications after UGI surgery and are associated with a high mortality rate [2, 3, 12]. The endoscopic management of these life-threatening complications has been described as a safe and feasible alternative to surgery [2, 13, 14] and, over the last 10 years, self-expandable stent (SES) placement has been the most commonly performed endoscopic procedure. More recently, E-VAC has been proposed as a different option to treat perforations after UGI surgery. Two recent metanalysis compared the effectiveness of SES and E-VAC for the treatment of UGI leaks and perforations [5, 10]. Both evidenced a significantly higher healing rate in E-VAC patients, with a shorter treatment duration and a reduced incidence of major complications and in-hospital mortality.

In the present study, we reported our initial experience with E-VAC therapy. Over a period of about two years, we treated 7 patients with E-VAC therapy, obtaining a complete healing of the leak/perforation, without need of further treatments, in 5 of them. Of note, in one patient, intraluminal E-VAC placement was unsuccessful, while a subsequent intracavitary treatment obtained a complete healing of the leak. Nevertheless, compared with the other experience published on E-VAC [15, 16] that reported a median healing time of 12–14 days (with a median of 3–5 sponges/patient), we observed a longer duration of the treatment (median duration: 41 days; median number of sponges per patient: 10). A possible explanation for this difference is that in our series E-VAC was always used as a secondary treatment. Median interval time between leak detection and start of E-VAC was 30 days, considerably longer than the interval of 3 days reported by Min [15] and the immediate placement of the sponge at leak detection reported by Berlth [16]. It is, therefore, possible that we treated with E-VAC only severe leaks that were refractory to other types of management. Another possible explanation for the longer duration of our treatments might be the inferior value of negative pressure that we applied to the sponge compared to other case series. As the device currently commercialized in our Country does not include a vacuum pump, we used a Pleure-Vac system with a negative pressure value of about 35 mmHg. The decision was motivated by the concerns we had of applying elevated negative pressure (a negative pressure up to 125 mmHg is possible according to the indications of the company which commercializes the product) in a delicate structure as an anastomosis or a gastric conduit, often located near the major vessels and airways. The success rate of 71% we observed might indicate, however, that even with less negative pressure, the E-VAC can be effective at the price of longer healing time.

Our findings confirmed that E-VAC therapy is a safe option for leak management. During the 60 procedures, we observed only one complication, a bleeding that was controlled endoscopically. The risk of bleeding during E-VAC treatment was already described by Laukoetter [17], who in his series on 52 patients and 390 sponge substitutions reported 5 minor bleedings (1,3%) managed endoscopically and 2 fatal hemorrhages. Moreover, E-VAC therapy confirmed to be well tolerated by the patients, even in longer treatments: in our experience, only one patient with a cervical leak interrupted the therapy after 4 days for neck and head pain.

Overall, we reported 6 cases of intracavitary and 2 intraluminal placements of the sponge. To date, there is no agreement on which is the best option [18], although the intraluminal approach is usually proposed in the absence of a large cavity. When a cavity is present, the intraluminal sponge placement has a reduced effectiveness in draining the collection and often requires percutaneous drain placement, thus reducing the theoretical advantages of E-VAC compared with SES. Moreover, in case of small wall defect associated with a large cavity, the device can act as a plug, thus resulting in an exclusion of a non-drained collection [19]. In these situations, it has been suggested to dilate the wall defect to allow the intracavitary placement of the device [20], as we did in one of our cases with success.

The present study has several limitations. First, it is a small retrospective study; hence, a selection bias is possible. Second, our cohort was heterogeneous, as we included patients with different operations and leak site.

## Conclusion

In conclusion, our study confirms E-VAC therapy to be a safe and effective option for the management of refractory leaks and perforations after UGI surgery, even if it requires longer treatment duration. Further studies are required to assess whether it should be the preferred first-line treatment and which is the optimal timing, the best site of sponge placement and negative pressure that should be applied.
